# Transcriptomic Insights into Post-Spawning Death and Muscle Atrophy in Ayu (*Plecoglossus altivelis*)

**DOI:** 10.3390/ijms26020434

**Published:** 2025-01-07

**Authors:** Jiancheng Liang, Minoru Fujisawa, Shogo Toma, Shuichi Asakawa, Kazutoshi Yoshitake, Yoji Igarashi, Shunsuke Saito, Takashi Akutsu, Kyuma Suzuki, Shigeharu Kinoshita

**Affiliations:** 1 Graduate School of Agricultural and Life Sciences, The University of Tokyo, Bunkyo 113-8657, Tokyo, Japan; liang-jian-cheng263@g.ecc.u-tokyo.ac.jp (J.L.); brmi17141@g.nihon-u.ac.jp (M.F.); tanapy666@gmail.com (S.T.); asakawa@g.ecc.u-tokyo.ac.jp (S.A.); 2 School of Marine Biosciences, Kitasato University, Sagamihara 252-0373, Kanagawa, Japan; akyoshita@g.ecc.u-tokyo.ac.jp; 3 Graduate School of Bioresources, Mie University, 1577 Kurima-machiya, Tsu 514-8507, Mie, Japan; igarashi@bio.mie-u.ac.jp; 4 Gunma Prefectural Fisheries Experiment Station, 13 Shikishima, Maebashi 371-0036, Gunma, Japan; saito-sh@pref.gunma.lg.jp (S.S.); akutsu-t@pref.gunma.lg.jp (T.A.); suzuki-q@pref.gunma.lg.jp (K.S.)

**Keywords:** *Plecoglossus altivelis*, spawning, skeletal muscle, RNA-seq, AP-1 TF family, muscle atrophy

## Abstract

In semelparous species like the ayu (*Plecoglossus altivelis*), spawning is followed by rapid physiological decline and death; yet, the underlying molecular mechanisms remain largely unexplored. This study examines transcriptomic changes in ayu skeletal muscle before and after spawning, with a focus on key genes and pathways contributing to muscle atrophy and metabolic dysfunction. Through RNA sequencing and DEG analysis, we identified over 3000 DEGs, and GSEA and KEGG pathway analysis revealed significant downregulation of energy metabolism and protein degradation. In post-spawning ayu, a rapid decrease in body weight was observed, accompanied by a decline in the expression of myosin heavy chain genes, which are major muscle protein genes, and gene expression changes indicative of muscle atrophy. Decreased expression of AP-1 transcription factors associated with muscle development and aging was also evident. PPI network analysis identified carbohydrate catabolism protein gapdh may be the key factor that led to muscle atrophy and accelerated aging in ayu. Our study revealed that after spawning, the ayu muscle tissue undergoes strong metabolic disorders and cellular stress responses, providing special insights into the mechanisms through the post-spawning death of ayu.

## 1. Introduction

The ayu *(Plecoglossus altivelis)* belongs to the order Osmeriformes and the family Plecoglossidae. It is widely distributed in Asian countries, including Japan, China, Korea, and Vietnam, and is an important economic fish species [1,2,3]. Ayu exhibits two distinct life history patterns: amphidromous and landlocked forms. The amphidromous form spends its larval stage in marine waters before migrating upstream in spring for growth, followed by downstream migration for spawning in autumn. The landlocked form resides primarily in Lake Biwa, Japan, spending its entire life cycle in freshwater [4,5,6].

Ayu is characterized as a short-lived fish species, with most individuals in natural populations dying shortly after spawning, completing their life cycle within one year [7,8]. Similarly, artificially cultured ayu experience rapid deterioration and mortality following artificial reproduction. This post-spawning decline is accompanied by rapid weight loss, and most individuals die within 1–2 months.

In unstable or resource-limited environments, species often adopt fast-life-history strategies to ensure population persistence and development, characterized by rapid growth, early maturation, and high reproductive rates but short lifespans [9]. These organisms typically reproduce only once in their lifetime, followed by rapid senescence or death, a reproductive strategy known as semelparity [10]. The strategy of semelparity is common in plants and can also be observed in insects, but it is limited to a few species among vertebrates. Notable examples of semelparous vertebrates include Pacific salmon (*Oncorhynchus* spp.), where most mature individuals die after spawning in freshwater, with very few survivors, and the Australian dasyurid (*Dasyurus hallucatus*), where males die after mating, while females undergo exaggerated senescence [11,12].

Although studies have suggested that the rapid mortality in ayu may be associated with post-spawning hormonal homeostasis disruption, decreased appetite, or elevated levels of reactive oxygen species (ROS) [13,14], comparative experiments with caloric restriction failed to demonstrate any life-extending effects [15]. Research investigating the mechanisms underlying the rapid post-reproductive mortality in ayu remains limited, and the intrinsic mechanisms remain poorly understood.

Muscles are tissues that are significantly affected by aging. In mammals, muscle atrophy with aging, called sarcopenia, leads to a decline in physical abilities and a decrease in muscle-derived hormones, which in turn promotes overall aging. In fish, muscles grow throughout their lifespan, making muscle aging less apparent [16].

The activating protein-1 (AP-1) transcription factor (TF) family is a dimeric transcriptional complex involved in various cellular and physiological functions, comprising *Fos*, *Jun*, *Atf,* and *Maf* subfamily members [17,18]. Each member exhibits distinct expression and regulatory pathways, enabling the AP-1 TF family to participate extensively in processes such as cell proliferation, differentiation, and apoptosis. The AP-1 TF member is also involved in muscle differentiation and growth. Additionally, the AP-1 TF family is associated with aging. AP-1 TF family shows a decline in function in various tissues of aging mice, but their function is restored in calorie-restricted rejuvenated individuals. Our previous study indicated that the function of the AP-1 TF family is maintained in the muscles of aging zebrafish [19], suggesting that this may contribute to the anti-aging properties of fish muscles. However, it is intriguing to consider whether muscle aging occurs in ayu, which die rapidly after spawning, and how the function of the AP-1 TF family changes in the lifespan characteristics of ayu.

Based on previous research and observations, we hypothesize that ayu undergo significant shifts in gene expression and stress responses after spawning. To test this hypothesis, we collected skeletal muscle samples from ayu before and after spawning and performed bulk RNA sequencing analysis. Through differential expression (DEG) and enrichment analysis, we present the first comprehensive transcriptomic study of ayu muscle tissue, identifying key genes and pathways potentially involved in post-spawning senescence and mortality in this species.

## 2. Results

### 2.1. Physical Decline and Transcriptomic Analysis Process in Spawning Ayu

**Figure 1:**
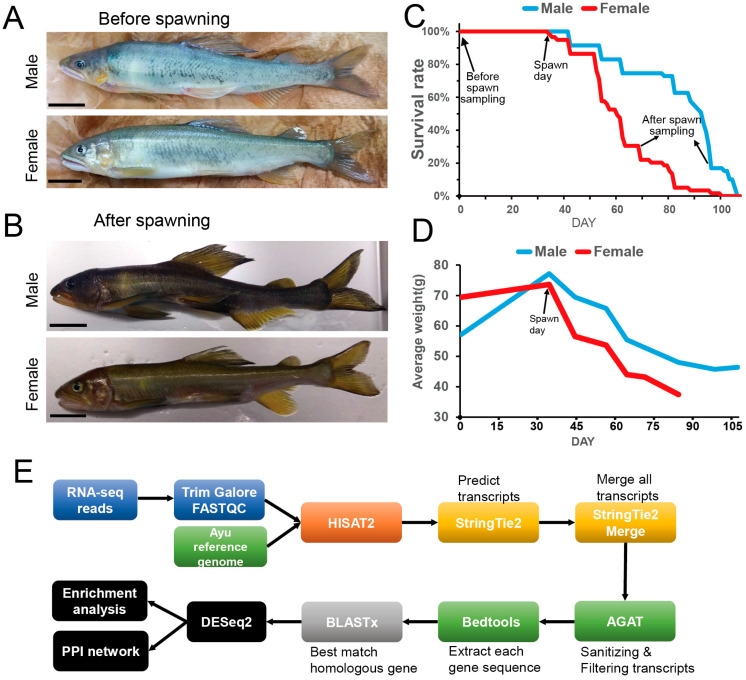
Ayu sample information and RNA-seq analysis method. (**A**,**B**) Changes in body size and external appearance of male and female ayu before and after spawning. Scale bar: 20 mm. (**C**) After spawning, the survival rate of ayu, with the x-axis representing the days and the y-axis showing the survival rate. Sampling was conducted as indicated by the black arrow. The before-spawn sampling was one month before the spawning day, and the after-spawn sampling was at approximately when more than half of the same-gender fish were dead. (**D**) Changes in body weight of ayu before and after spawning. (**E**) Schematic of RNA sequencing data analysis methods, providing an overview of the processing from raw RNA sequencing data process to subsequent result analysis.

Both male and female ayu maintained at the Gunma Prefecture Fisheries Experiment Station exhibited healthy characteristics before spawning, with robust body conditions, dark green coloration, and normal swimming behavior (Figure 1A). However, one month after spawning, ayu showed a significant physical decline, characterized by severe muscle atrophy and diminished body coloration (Figure 1B). Mortality rates increased progressively, with approximately half of the female population dying within one month after spawning and the remaining females dying gradually within two months.

Although male fish had lower mortality rates during the first month after spawning, their mortality rate surged in the second month, and they also died within approximately two–three months (Figure 1C). These patterns were consistently observed across four continuous breeding seasons from 2019 to 2022 under identical breeding conditions (Figure 1C and Figure S1A–C). However, we have recently observed that males have been dying earlier than females (Figure S1C). Therefore, the sex difference in the life span of ayu is not well understood. We also recorded the body weight changes of ayu before and after spawning (Figure 1D). In the month before spawning, both females and males gradually increased in weight, reaching a peak at the time of spawning. Spawning weight loss was particularly pronounced in females compared to males, with this declining trend continuing until death.

Therefore, we selected three pairs of pre-spawning and post-spawning ayu, totaling six females and six males, to be dissected approximately when more than half of the same gender fish are dead. We collected trunk skeletal muscle from the upper region near the pectoral fins, extracted mRNA, and performed bulk RNA sequencing. We utilized the recently published ayu reference genome [20] to map the RNA sequencing data to the genome and conducted transcriptome prediction across multiple samples. The predicted transcriptomes were merged, and redundancies were removed, followed by homologous gene identification using DIAMOND and subsequent DEG analysis. The complete analytical workflow is illustrated in (Figure 1E).

### 2.2. DEG Analysis Reveals Metabolic Decline and Protein Degradation in Ayu

**Figure 2:**
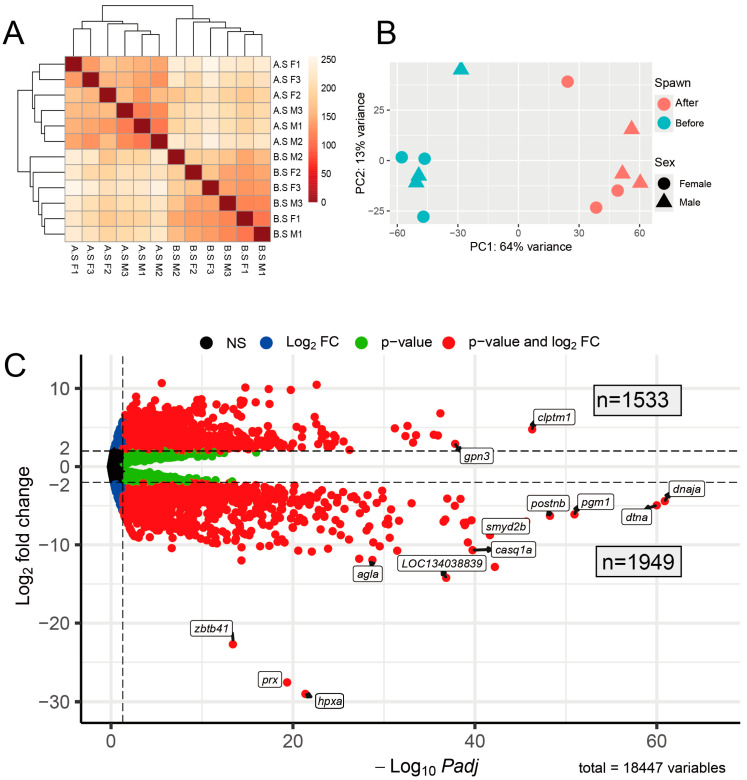
Cluster of ayu samples and DEGs. (**A**) Heatmap showing Euclidean distances between ayu samples. (A.S: after spawn. B.S: before spawn. F: female. M: male.) (**B**) Principal component analysis (PCA) plot of all samples’ variance based on VST normalized data. (**C**) Volcano plot illustrating DEGs in ayu samples from RNA-seq analysis. The numbers of upregulated and downregulated genes are displayed within light white boxes. Red dots represent genes identified as DEGs. Horizontal dashed lines denote genes with a fold change greater than twofold, while the vertical dashed line indicates the padj cutoff of 0.05.

Before performing DEG analysis, we assessed the variation between samples (Figure 2A). The results showed high similarity among the before-spawning (B.S) samples, with marked differences when compared to the after-spawning (A.S) samples, which also maintained strong internal consistency. These findings were supported by PCA plot analysis (Figure 2B), where PC1 accounted for 64% of gene expression variance and PC2 for 13%. Overall, these results clearly demonstrated the distinct differences between B.S and A.S skeletal muscle samples in ayu.

Subsequently, we conducted a DEG analysis and summarized the results in a volcano plot (Figure 2C) and table (Table 1). As expected, the number of downregulated DEGs (n = 1949) exceeded the number of upregulated DEGs (n = 1533) (Table S2). Notably, certain genes exhibited substantial fold changes, especially *zbtb41*, *prx*, and *hpxa*. *Hpxa* was first reported as a fish-specific protein *wap65*, which increases in the blood of goldfish acclimated to high temperatures [21]. It has been reported to be involved in the immune system and stress responses in several fish species [22]. In ayu, the levels of *wap65* mRNA and protein increase with high-temperature acclimation and cadmium exposure [23]. Its human homolog, hemopexin (*Hpx*), plays a crucial role in binding free heme to prevent heme-induced oxidative stress and inflammatory effects during conditions such as internal bleeding and rhabdomyolysis [24].

The significant decrease in *hpxa* in A.S ayu suggests a decline in stress and inflammatory responses in their bodies. Interestingly, the *prx* gene, which encodes the periaxin protein essential for maintaining peripheral nerve myelin, has been linked to Charcot–Marie–Tooth disease (CMT) in humans when mutated [25,26]. CMT is characterized by progressive muscle weakness and atrophy in distal limbs, which may be relevant to the muscle atrophy observed in A.S ayu.

**Figure 3:**
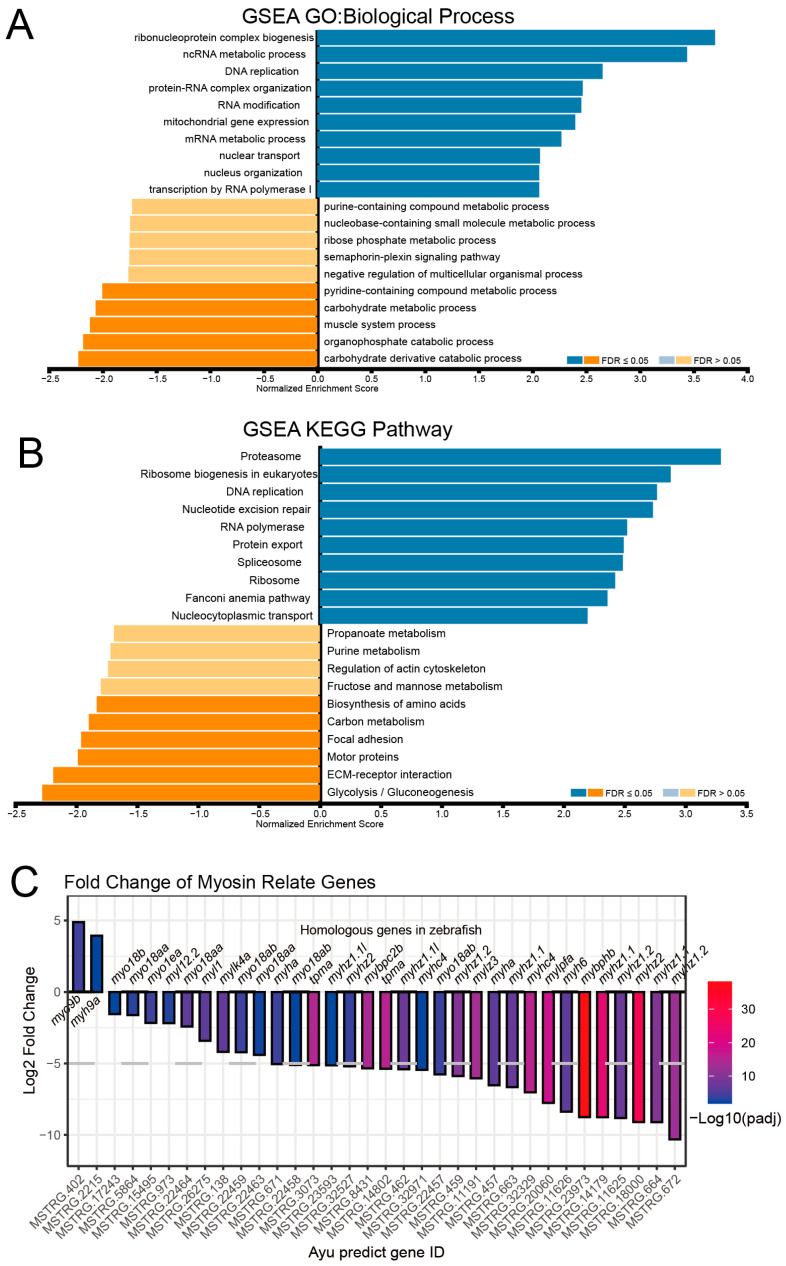
Gene ontology and pathway enrichment analysis of ayu DEGs. (**A**) GO enrichment of biological process. (**B**) KEGG pathway analysis of DEGs (FDR: false discovery rate; NES: normalized enrichment score; positive NES indicates upregulation, while negative indicates downregulation). (**C**) Fold change of myosin-related genes, with the x-axis labeling homologous gene names in zebrafish and the lower section (MSTRG.) indicating the corresponding gene ID numbers from transcriptome predictions. Color intensity reflects padj values.

Gene Set Enrichment Analysis (GSEA) of filtered DEGs revealed significant downregulation in catabolic and metabolic processes within the GO: Biological Process category (Figure 3A). Additionally, we also observed decreased activity in muscle system-related pathways, which aligns with our observed physical changes in A.S ayu. Interestingly, RNA metabolism and processing pathways were upregulated in A.S ayu, though the significance of this observation remains unclear (Figure S2). However, KEGG pathway analysis (Figure 3B) showed increased activity in proteasome-related pathways, which is consistent with muscle atrophy characteristics involving enhanced protein hydrolysis through the ubiquitin–proteasome pathway (UPP) and increased binding between ubiquitin and muscle proteins [27]. This elevated proteasome activity might be associated with the observed upregulation of RNA synthesis-related pathways. Notably, the glycolysis and carbon metabolism pathways—critical energy sources for skeletal muscle cells—were significantly downregulated in the KEGG results, indicating a substantial decrease in energy metabolism within ayu muscle tissue.

Furthermore, we investigated the changes in myosin-related proteins in DEGs (Figure 3C). Most myosin-related proteins showed substantial downregulation, especially genes associated with the myosin II complex within muscle fibers, such as *myhz1.1* and *myhz1.2*, which showed the largest decreases. Other proteins representing muscle contraction, like *myh6* and *myhc4*, also showed varying degrees of downregulation. Only *myh9a*, associated with cytoskeletal motor activity, and *myh9b*, related to microfilament motor activity, showed minor upregulation. These results suggest that post-spawning ayu experience a pronounced downregulation of energy metabolism pathways, an increase in proteasome-mediated protein degradation, and a decline in muscle-related skeletal proteins, which collectively may explain the body shrinkage and weight loss observed after spawning.

### 2.3. AP1 TF Family Undergo Downregulation After Spawning

The AP-1 TF family contributes to muscle cell differentiation and has been reported to show decreased expression and function in the aging process of mammals. Our previous research suggests that the expression of the AP-1 TF family is maintained in the muscles of zebrafish [19], which may contribute to the anti-aging properties of fish muscles.

**Figure 4:**
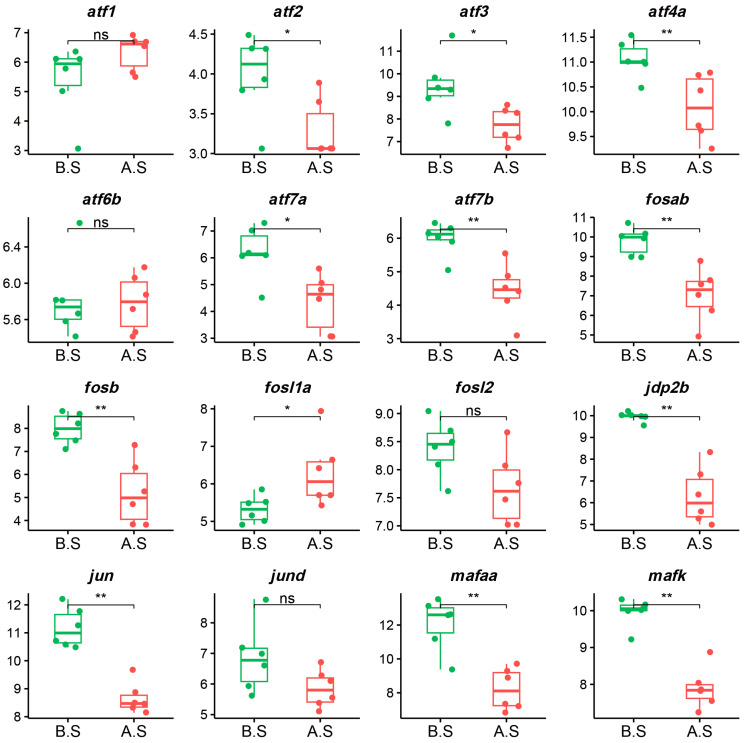
AP1 transcription factor expression level vst change. Comparison of vst-normalized counts of the AP1 transcription factor family in ayu before and after spawning, with the x-axis labels “A.S” representing after spawning and “B.S” representing before spawning and the y-axis showing vst-normalized counts. Values are presented as means ± SD, with statistical significance assessed via the Mann–Whitney u test. (* *p*  <  0.05, ** *p*  <  0.01, ns: not significant).

Consequently, we investigated the expression levels of the AP-1 TF family in the ayu’s skeletal muscle, identifying 22 AP-1 TF expressed in the ayu transcriptome (Figure 4 and Table S1). Among them, 11 factors were downregulated, only 1 factor was upregulated, and the remaining 10 showed no significant changes. Notably, the *fosab* and *fosb* proteins, which are crucial for initiating regeneration programs in skeletal muscle stem cells [28,29], were significantly downregulated. Additionally, the ATF TF family, which is involved in muscle biology and influences various processes and metabolic regulation, also displayed relevant changes. For instance, *atf2*, which is considered an activator of the Wnt/Ca2+ signaling pathway [30], was downregulated in our data. *Atf3*, known to function as either a transcriptional repressor or activator depending on the cellular context [31,32], also showed a tendency toward downregulation. Among the upregulated factors, *fosl1a* is associated with various cellular differentiation processes, oxidative stress, and inflammatory processes [33]. Overall, our data offer insights into changes within the AP-1 TF family in ayu post-spawning, providing clues about the dramatic physiological shifts experienced after spawning.

### 2.4. GAPDH May Be the Major Regulator of Ayu Infirmity

**Figure 5:**
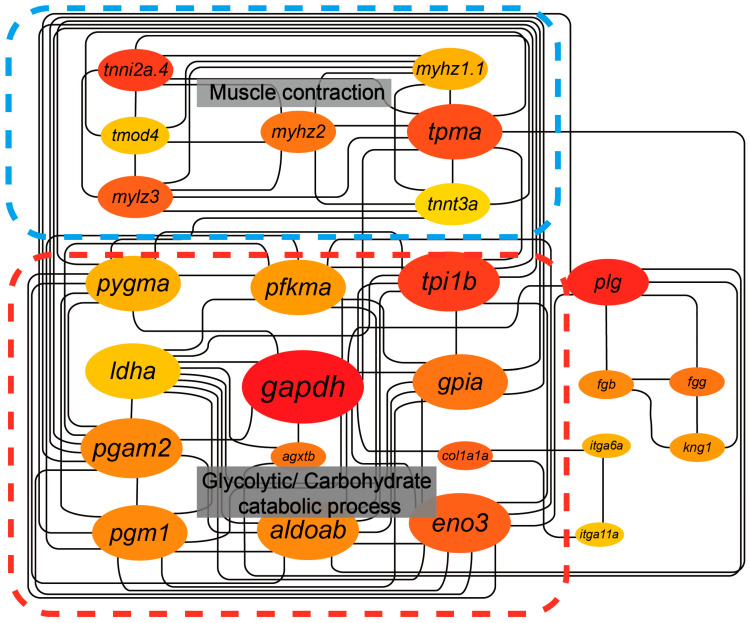
PPI network of ayu DEGs. PPI network showing various proteins in ayu DEGs, where each node connection represents a potential interaction. The top 25 most significant protein nodes among DEGs, calculated using the degree algorithm in CytoHubba, are represented by larger and darker-colored circles to indicate higher scores.

Finally, we performed Protein–Protein Interaction (PPI) network analysis of the DEGs to identify key factors, core pathways, and possible molecular mechanisms underlying the drastic physiological changes observed in ayu A.S. By screening for hub proteins with numerous protein connections (Figure 5), we identified *gapdh* as the most critical node protein, followed by *plg*, *tpi1b*, *tnni2a.4*, *tpma*, and *eno3*.

All of these proteins are associated with the glycolytic process and carbohydrate catabolism, and they were significantly downregulated after spawning, suggesting inhibited glycolysis and disrupted cellular energy metabolism in ayu muscle. Insufficient ATP supply can impair normal muscle cell contraction, leading to myosin degradation and muscle atrophy. On the other hand, the gapdh-catalyzed reaction involves NAD^+^ reduction to NADH, potentially causing NAD^+^/NADH ratio imbalance [34]. This further weakens cellular antioxidant capacity, facilitating reactive oxygen species accumulation and potentially inducing cellular senescence or apoptosis. The PPI network also highlighted proteins involved in muscle contraction, such as *myhz*, *mylz*, *tpma*, etc., which were also downregulated in the DEG analysis.

Combined with the observed disruptions in cellular energy metabolism, these findings suggest that A.S ayu experience nutritional deficiencies or energy depletion, activating pathways that degrade muscle proteins and resulting in reducing muscle fiber size or number, ultimately causing muscle atrophy.

## 3. Discussion

It is unclear whether the rapid physiological decline and death of ayu after spawning is due to the rapid progression of aging. For example, while calorie restriction can inhibit aging and extend lifespan in many organisms, it does not have a lifespan-extending effect on ayu after spawning [15]. The results from our transcriptomic analysis of ayu skeletal muscle before and after spawning, which reveal changes such as the decline in stress response and immune function indicated by hemopexin changes, decreased expression of the AP-1 TF family, and muscle atrophy in post-spawning ayu, are similar to the aging process of mammalian skeletal muscles. Nagasaka et al. [35] also demonstrated elevated DNA damage and the phosphorylation of *p53* in the brain of ayu after spawning. Combined with our results, these findings suggest that the rapid death of ayu after spawning shares some commonalities with the aging process in mammals. Furthermore, the decline in AP-1 expression in ayu after spawning may increase the susceptibility of muscle cells to oxidative stress and other damaging factors, potentially leading to cellular dysfunction and tissue atrophy. This observation aligns with changes in AP-1 activity observed during muscle aging in mammals, suggesting that AP-1 downregulation may be a pivotal event in the muscle atrophy process [36,37,38].

On the other hand, the influence of the stress hormone cortisol on the post-spawning death of fish has also been suggested. Cortisol is particularly crucial, representing the most important hormonal regulatory mechanism in fish, combining the functions of both glucocorticoids and mineralocorticoids observed in mammals [39,40,41]. Cortisol influences fish metabolism of carbohydrates, proteins, and lipids, primarily by mobilizing energy reserves through glycolysis and gluconeogenesis, ensuring sufficient energy supply to support reproductive activities [42]. In fish, cortisol levels in the blood increase during maturation and then return to normal levels. However, in the Atlantic salmon, a representative semelparous fish, cortisol levels remain elevated even after spawning and subsequent decline [43]. Prolonged cortisol stimulation can lead to severe energy depletion. Cortisol can induce muscle atrophy by degrading proteins through the ubiquitin–proteasome system and autophagy–lysosome pathway [44]. Elevated cortisol levels also increase cellular oxidative stress, inducing apoptosis across various cell types causing DNA damage and cellular senescence [45]. Although ayu and pacific salmon belong to different orders within the class Actinopterygii, with ayu classified under Osmeriformes and salmon under Salmoniformes, both species follow this semelparous reproductive strategy. However, current research on cortisol hormone secretion and concentration in ayu before and after spawning remains limited.

Our PPI network analysis revealed that the function of the potentially critical protein network, centered around *gapdh*, involved in glycolytic and carbohydrate catabolic processes, declines in ayu skeletal muscle after spawning. Dysfunction of these protein networks, including *gapdh* causes abnormal energy metabolism in the muscles, resulting in a decline in muscle function. Additionally, it has become clear that *gapdh*, in addition to its role as a glycolytic enzyme, also plays roles in stress resistance, cell death, and DNA repair [34]. Dysfunctions in these roles are directly linked to aging. While a direct relationship between cortisol and *gapdh* is not known, the NAD^+^/NADH ratio imbalance caused by *gapdh* metabolic dysfunction can also be observed with cortisol excess in mice [46]. The synergistic effect of metabolic abnormalities caused by excess cortisol and the dysfunction of the *gapdh* cascade may influence the rapid aging and death of semelparous fish after spawning.

While our findings are based on bioinformatic exploration, further biological experiments and validation are needed to support our hypotheses. Moreover, our study is based on skeletal muscle tissues; other tissues during spawning remain to be elucidated. Nevertheless, our study provides valuable data for future investigations into the spawning aging and mortality of ayu, contributing to the broader understanding of semelparous reproduction in fish species.

## 4. Materials and Methods

### 4.1. Sample Collection

All animal experimentations were in accordance with the University of Tokyo Animal Experiment Implementation Manual. Farmed ayu were sampled from the Gunma Prefecture Fisheries Experiment Station in Japan Gunma Prefecture.

On 31 July 2019, approximately one month prior to the spawning season of ayu, we collected 3 females and 3 males from an artificially bred group as the before spawning samples. Following artificial insemination and egg collection on September 3, 59 males and 59 females were cultivated in FRP tanks (4.0 × 1.5 × 0.9 m) with recirculating system in large outdoor water and temperatures maintained at approximately 17–19 °C. The fish were provided with daily feedings, and mortalities were recorded until all individuals had died. Additionally, regular measurements were conducted to monitor the body weight of ayu.

Given the observed differences in post-spawning survival rates between males and females, post-spawning sampling was performed when more than half of each gender’s population had died. Specifically, three females were sampled on October 3 (31 days post-spawning), and three males were sampled on November 6 (65 days post-spawning), as shown in Figure 1C.

The collected ayu individuals were dissected locally immediately following euthanasia via decapitation. Skeletal muscle tissue samples were extracted from the upper trunk region near the pectoral fins. The tissue samples were promptly immersed in NucleoProtect RNA (Takara Bio, Shiga, Japan) solution, stored at −30 °C, and used for mRNA extraction within one week.

### 4.2. Preparation of cDNA Library and Sequencing

Total RNA was extracted from the collected tissue samples using TRIzol ( Invitrogen, Carlsbad, CA, USA according to the manufacturer’s protocol. All extracted RNA samples were treated with DNase I (Takara Bio, Shiga, Japan) to remove residual DNA. The integrity of RNA was assessed on Agilent 2100 TapeStation using RNA ScreenTape(Agilent, Santa Clara, CA, USA). For library construction, samples collected before spawning with RIN values > 7 were selected. Due to the challenging cellular and tissue environment for RNA stability in after-spawning ayu, as they are typically subject to muscle atrophy and increased mortality risks, after-spawning samples with RIN values > 6 were used for subsequent library construction. Strand-specific sequencing libraries were prepared using the Illumina Stranded mRNA Prep Kit (Illumina, San Diego, CA, USA) with slight modifications to the manufacturer’s protocol. Library size and concentration were assessed on an Agilent 2100 TapeStation using D1000 ScreenTape(Agilent, Santa Clara, CA, USA). Finally, the libraries were pooled and sequenced on the Illumina HiSeq X system.

### 4.3. RNA Sequencing Data Process

Quality control and sequence filtering of the RNA raw read files were conducted using Trimgalore (version 0.6.10) to remove duplicates, low-complexity sequences, and low-quality reads, with the following options: --paired--illumina--stringency 5--quality 25--length 25. The processed reads were then examined using FASTQC to ensure quality. The ayu reference genome was obtained from the NCBI database (Assembly: ASM3657176v1, GenBank: GCA_036571765.1; accession number: PRJDB9085) [20]. Sample reads were aligned to this reference genome using HISAT2 (version 2.2.1) [47], and alignments were sorted and converted to BAM format with Samtools (version 1.17) [48]. The expression matrix can be found in Supplementary Materials (Table S3).

Transcript assembly was performed for each sample using StringTie2 (version 2.1.7) [49], and assembled transcripts from all samples were subsequently merged using the stringtie--merge command. Finally, the merged transcripts were validated, corrected, and supplemented with gene features and attributes using the AGAT toolkit (version 1.4.2) [50], resulting in a comprehensive genome annotation file specifying gene locations and functional regions for downstream analyses.

For gene quantification, StringTie2 was used to quantify gene expression levels based on the merged and corrected genome annotation file. Raw counts for each gene across samples were extracted using the prepDE.py script provided with StringTie2, generating an expression matrix for downstream DEG analysis.

### 4.4. Identification of Homologous Genes and Functional Annotation in Ayu

First, ayu gene sequences were extracted from the processed genome annotation file using the BEDTools getfasta command (version 2.28.0) [51]. A protein database was then constructed by downloading the nonredundant (NR) protein database from the NCBI database, along with all annotated protein sequences of species within the order Osmeriformes (freshwater smelts and related species). DIAMOND (version 2.1.8) [52] was used to search for homologous genes in this database that showed high similarity to the ayu gene sequences. The best match result for each sequence was selected using JCVI toolkit (version 1.4.21) [53] with the command “jcvi.formats.blast best” to obtain the optimal alignment results. Functional annotation of ayu genes was then performed based on the best alignment results.

However, as the best alignment results may include uncharacterized or functionally ambiguous proteins, which could hinder downstream enrichment and protein interaction network analyses, we also conducted homology searches in the zebrafish (*Danio rerio*) protein database (GRCz11) [54] to identify homologous ayu genes. The best matches from this zebrafish database were selected for use in subsequent pathway enrichment and protein network analyses.

### 4.5. DEG, Enrichment, and PPI Network Analysis

DEG analysis between the pre- and post-spawning groups was conducted using the DESeq2 software (version 1.44.0) [55]. Genes/transcripts were excluded from analysis if the number of samples with a raw count value greater than 10 was less than the minimum group size. In this experiment, the minimum group size was set to 3. Genes/transcripts with an adjusted *p*-value below 0.05 and |log fold change| ≥ 2 were considered DEGs. The variance stabilizing transformation (VST) method was applied to normalize gene/transcript expression levels. For multiple transcripts that have the same blast result/gene name, we took the average for subsequent statistics.

For visualization, Euclidean distances between all samples were calculated using the dist function in R, and heatmaps were generated using the pheatmap package. PCA analysis was performed with the plotPCA function in DESeq2, and plots were generated using ggplot2. The EnhancedVolcano package (version 1.22.0) [56] was used to create a volcano plot of DEGs, with cutoffs set at padj < 0.05 and log fold change ≥ 2.

Subsequently, GSEA analysis was performed using the WebGestalt platform [57], including gene ontology (GO) and KEGG pathway enrichment analyses. For the PPI network analysis, 480 proteins were selected based on |log fold change| ≥ 5 and *p*-value < 0.01 in DEG result. The STRING platform [58] was used to construct a PPI network, with the interaction score set to medium confidence and isolated nodes hidden in the network. The original network was then refined in Cytoscape software (version 3.10.1), where the MCODE and CytoHubba plugins were used to determine interactions among the key genes within the network. Key genes were identified based on five distinct computational methods: EPC, MCC, DMNC, MNC, and stress.

## 5. Conclusions

In this study, we conducted a transcriptomic analysis to investigate the gene expression changes underlying post-spawning mortality in ayu. Our RNA-seq results revealed that, after spawning, ayu muscle tissue experiences severe metabolic disorders and cellular stress responses, including significant downregulation of myosin-related proteins and most AP-1 transcription factors. Additionally, PPI network analysis identified key proteins potentially involved in post-spawning mortality, with gapdh emerging as a critical node. Taken together, our research provides valuable insights into the mechanisms through the post-spawning death of ayu, and the findings hold the potential to inform investigations for ayu physiological change and muscle atrophy, which could benefit the ayu cultivation and production.

## Figures and Tables

**Table 1:** Top 10 upregulated or downregulated genes after spawning.

Predict ID	Log2FC	Gene Symbol	Gene Annotation	Padj	E.Value
Upregulated Genes					
MSTRG.19953	10.67146066	*PYYA*	Peptide YY-A	3.09265 × 10^−5^	3.4 × 10^−26^
MSTRG.2265	10.46140617	*FAM161A*	Protein FAM161A	5.03903 × 10^−21^	1.8 × 10^−26^
MSTRG.23550	10.1078415	*PTGIS*	Prostacyclin synthase	1.17564 × 10^−13^	1.2 × 10^−45^
MSTRG.12358	9.914623448	*NR0B2B*	Nuclear receptor subfamily 0, group B, member 2b	4.72287 × 10^−16^	6.2 × 10^−148^
MSTRG.3709	9.800333007	*TPH1A*	Tryptophan 5-hydroxylase 1a	2.42473 × 10^−18^	7.5 × 10^−28^
MSTRG.4436	8.936884854	*LOC136948060*	Forkhead box protein N3-like	0.008114325	5.2 × 10^−75^
MSTRG.13415	8.617214914	*LOC124485982*	Desmin-like	3.68045 × 10^−09^	2.2 × 10^−140^
MSTRG.8139	8.47117737	*ANKRD1B*	Ankyrin repeat domain-containing protein 1b	0.00013683	1.3 × 10^−27^
MSTRG.24563	8.447603809	*LOC136945842*	Carbonic anhydrase 6-like	3.44952 × 10^−13^	2.8 × 10^−111^
MSTRG.30828	8.374425508	*MANSC1*	MANSC domain-containing protein 1	1.79458 × 10^−13^	4.5 × 10^−44^
Downregulated Genes					
MSTRG.25731	−29.02395479	*HPXA, WAP65*	Warm temperature acclimation 65 kDa protein 1	7.77379 × 10^−20^	3.9 × 10^−40^
MSTRG.6202	−27.56227049	*PRX*	Neuroblast differentiation-associated protein AHNAK	5.95638 × 10^−18^	0
MSTRG.318	−22.71584661	*ZBTB41*	Zinc finger and BTB domain-containing protein 41	2.19762 × 10^−12^	7 × 10^−116^
MSTRG.17263	−14.21396383	*LOC134038839*	Uncharacterized protein LOC134038839	1.35903 × 10^−34^	0
MSTRG.7386	−12.82401618	*LOC136954605*	Carnosine synthase 1-like	1.65443 × 10^−39^	0
MSTRG.15201	−11.9962021	LOC136955384	Mucin-2	2.97838 × 10^−13^	0
MSTRG.18085	−11.93369054	*LOC124473927*	Dual specificity phosphatase 29-like	7.80727 × 10^−27^	1.8 × 10^−41^
MSTRG.1275	−11.78117942	*AGLA*	Glycogen debranching enzyme	1.89853 × 10^−25^	9.9 × 10^−31^
MSTRG.16761	−10.86620076	*GAPDH*	Glyceraldehyde-3-phosphate dehydrogenase	1.65317 × 10^−20^	1.5 × 10^−36^
MSTRG.25027	−10.74879933	*OGNB*	Osteoglycin, paralog b	2.01811 × 10^−29^	1.3 × 10^−42^

Log2FC: log2Foldchange, E.value: BLAST E.value.

## Data Availability

RNA-seq data of ayu generated and analyzed in this study are available in the DDBJ, BiopProject Accession PRJDB19565. at https://ddbj.nig.ac.jp/search/entry/bioproject/PRJDB19565 (accessed on 14 November 2024).

## Supplementary Materials

The following supporting information can be downloaded at https://www.mdpi.com/article/10.3390/ijms26020434/s1.
