# Supplementary material for: Transcriptomic Insights into Post-Spawning Death and Muscle Atrophy in Ayu (Plecoglossus altivelis)
Source: Int J Mol Sci. 2025 Jan 7;26(2):434. doi: 10.3390/ijms26020434 (PMC11764881; doi:10.3390/ijms26020434)
Supplement: Supplementary file 1 [file ijms-26-00434-s001.zip › Supplementary FigureS1 S2.pdf]

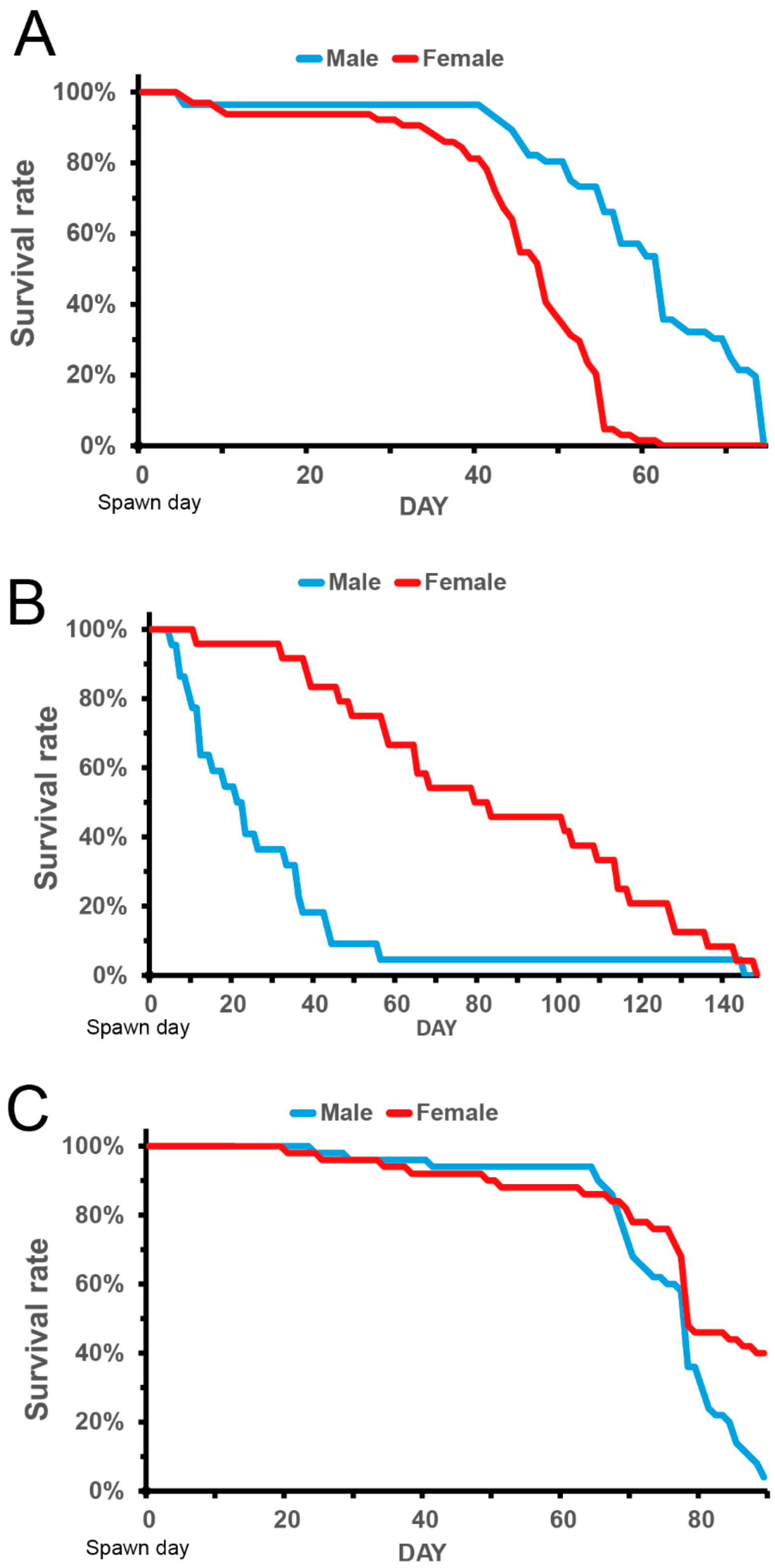

Figure S1. Survival plot of Ayu following spawning across different years (A: 2020, B: 2021, C: 2022), with the x-axis representing days of post-spawning and the y-axis showing the survival rate.

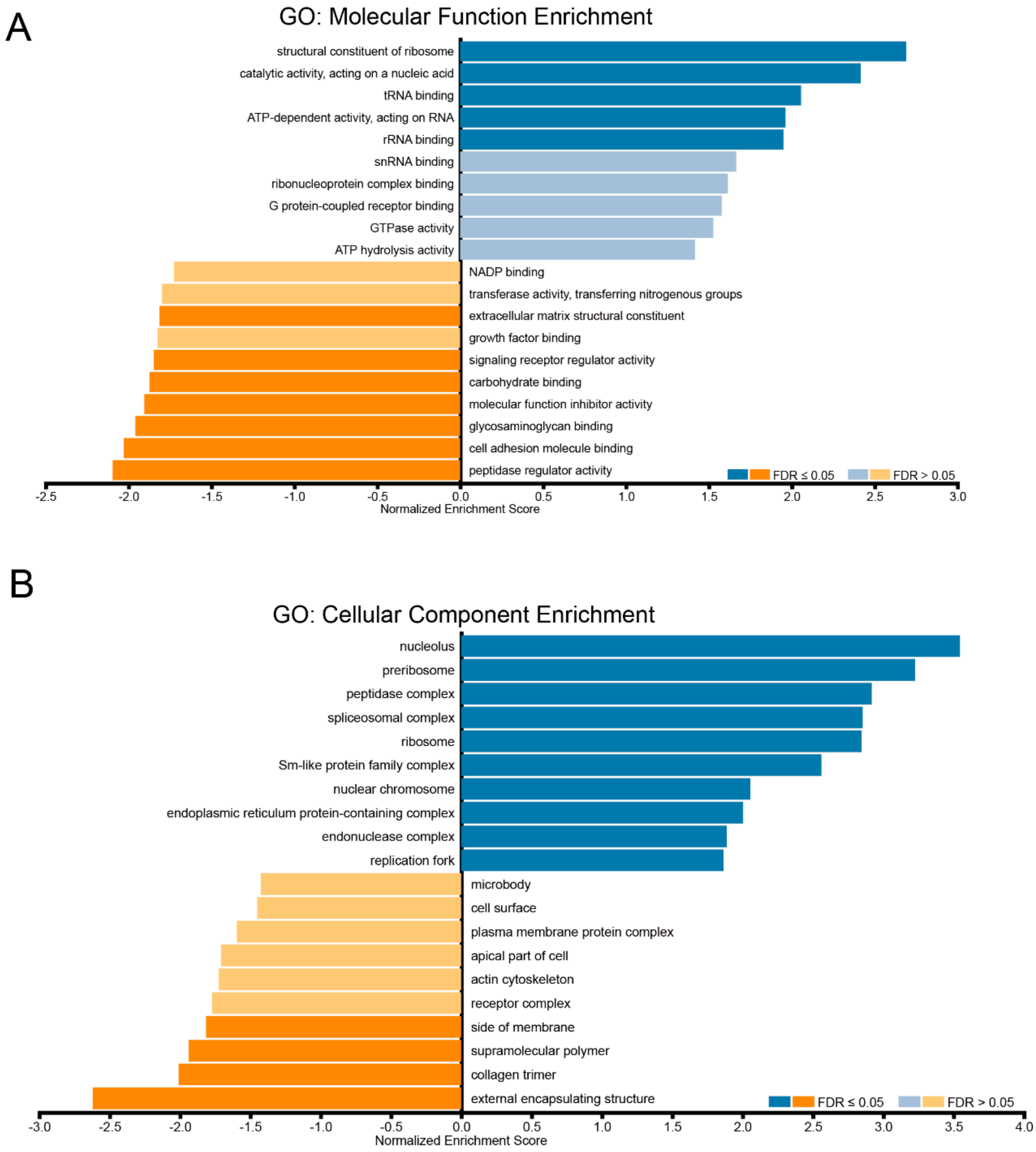

Figure S2. Gene ontology and pathway enrichment analysis of Ayu DEGs. (A) GO enrichment: Cellular component analysis. (B) GO enrichment: Molecular function analysis. (FDR: false discovery rate, NES: normalized enrichment score.
